# Impact of interventions on the incidence of natural focal diseases during the outbreak of COVID-19 in Jiangsu Province, China

**DOI:** 10.1186/s13071-021-04986-x

**Published:** 2021-09-19

**Authors:** Xiaoqing Cheng, Jianli Hu, Li Luo, Zeyu Zhao, Nan Zhang, Mikah Ngwanguong Hannah, Jia Rui, Shengnan Lin, Yuanzhao Zhu, Yao Wang, Meng Yang, Jingwen Xu, Xingchun Liu, Tianlong Yang, Weikang Liu, Peihua Li, Bin Deng, Zhuoyang Li, Chan Liu, Jiefeng Huang, Zhihang Peng, Changjun Bao, Tianmu Chen

**Affiliations:** 1 Jiangsu Provincial Center for Disease Control and Prevention (Jiangsu Institution of Public Health), Nanjing, 210009 Jiangsu People’s Republic of China; 2 State Key Laboratory of Molecular Vaccinology and Molecular Diagnostics, School of Public Health, Xiamen University, Xiamen, 361102 Fujian People’s Republic of China; 3 Medical College, Xiamen University, Xiamen, 361102 Fujian People’s Republic of China; 4 School of Public Health, Nanjing Medical University, Nanjing, 211166 Jiangsu People’s Republic of China

**Keywords:** COVID-19, Natural focal diseases, Impact, Intervention

## Abstract

**Background:**

During the period of the coronavirus disease 2019 (COVID-19) outbreak, strong intervention measures, such as lockdown, travel restriction, and suspension of work and production, may have curbed the spread of other infectious diseases, including natural focal diseases. In this study, we aimed to study the impact of COVID-19 prevention and control measures on the reported incidence of natural focal diseases (brucellosis, malaria, hemorrhagic fever with renal syndrome [HFRS], dengue, severe fever with thrombocytopenia syndrome [SFTS], rabies, tsutsugamushi and Japanese encephalitis [JE]).

**Methods:**

The data on daily COVID-19 confirmed cases and natural focal disease cases were collected from Jiangsu Provincial Center for Disease Control and Prevention (Jiangsu Provincial CDC). We described and compared the difference between the incidence in 2020 and the incidence in 2015–2019 in four aspects: trend in reported incidence, age, sex, and urban and rural distribution. An autoregressive integrated moving average (ARIMA) (*p*, *d*, *q*) × (*P*, *D*, *Q*)_*s*_ model was adopted for natural focal diseases, malaria and severe fever with thrombocytopenia syndrome (SFTS), and an ARIMA (*p*, *d*, *q*) model was adopted for dengue. Nonparametric tests were used to compare the reported and the predicted incidence in 2020, the incidence in 2020 and the previous 4 years, and the difference between the duration from illness onset date to diagnosed date (DID) in 2020 and in the previous 4 years. The determination coefficient (*R*^2^) was used to evaluate the goodness of fit of the model simulation.

**Results:**

Natural focal diseases in Jiangsu Province showed a long-term seasonal trend. The reported incidence of natural focal diseases, malaria and dengue in 2020 was lower than the predicted incidence, and the difference was statistically significant (*P* < 0.05). The reported incidence of brucellosis in July, August, October and November 2020, and SFTS in May to November 2020 was higher than that in the same period in the previous 4 years (*P* < 0.05). The reported incidence of malaria in April to December 2020, HFRS in March, May and December 2020, and dengue in July to November 2020 was lower than that in the same period in the previous 4 years (*P* < 0.05). In males, the reported incidence of malaria in 2020 was lower than that in the previous 4 years, and the reported incidence of dengue in 2020 was lower than that in 2017–2019. The reported incidence of malaria in the 20–60-year age group was lower than that in the previous 4 years; the reported incidence of dengue in the 40–60-year age group was lower than that in 2016–2018. The reported cases of malaria in both urban and rural areas were lower than in the previous 4 years. The DID of brucellosis and SFTS in 2020 was shorter than that in 2015–2018; the DID of tsutsugamushi in 2020 was shorter than that in the previous 4 years.

**Conclusions:**

Interventions for COVID-19 may help control the epidemics of natural focal diseases in Jiangsu Province. The reported incidence of natural focal diseases, especially malaria and dengue, decreased during the outbreak of COVID-19 in 2020. COVID-19 prevention and control measures had the greatest impact on the reported incidence of natural focal diseases in males and people in the 20–60-year age group.

**Graphical Abstract:**

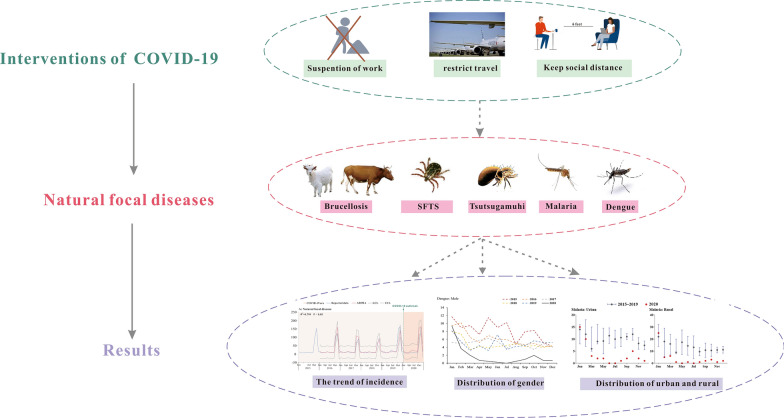

## Background

The coronavirus disease 2019 (COVID-19) pandemic spread rapidly worldwide, posing a formidable threat to global public health [1, 2]. The movement of people between Jiangsu Province and Hubei Province led to a massive burden in controlling the COVID-19 epidemic in Jiangsu Province [3]. The first confirmed case of COVID-19 was found in Jiangsu Province on January 22, 2020 [4], soon followed by an outbreak of 729 cases as of June 10, 2021. The government actively responded to the outbreak of COVID-19 with countermeasures including lockdown, travel restrictions and suspension of work [5, 6].

Several studies have shown that corresponding prevention and control measures, including travel restrictions, restricting population movement and suspension of work, were effective in controlling the spread of COVID-19 [7–9]. However, these measures also influenced the surveillance and spread of other infectious diseases [10, 11], including natural focal diseases such as malaria and dengue [12, 13]. A study in Japan showed that seasonal influenza activity in the country after the COVID-19 outbreak in 2020 was lower than in previous years [14]. However, social distancing led to an increase in dengue cases in Thailand [15]. Therefore, the impacts of COVID-19 interventions on the spread of other infectious diseases remain inconsistent.

The autoregressive integrated moving average (ARIMA) model is a tool to predict the trend of infectious diseases [16, 17], In this study, we established an ARIMA model based on the reported incidence of natural focal diseases from 2015 to 2019, and predicted the incidence in 2020. Next, we compared the predicted incidence with the reported incidence and analyzed the impact of COVID-19 prevention and control measures in Jiangsu Province. In this study we aimed at providing scientific evidence for the prevention and control of natural focal diseases during COVID-19.

## Methods

### Study area

Jiangsu Province is located in eastern China, with an area of 40,000 square miles and a population of about 80 million (the fifth most populated amongst the provinces in China). As of December 31, 2020, Jiangsu Province had more than 600 confirmed cases of COVID-19, and several studies reported the presence of different types of natural focal diseases in Jiangsu Province [18–21]. At present, Jiangsu Province has not published an article about the impact of COVID-19 prevention and control measures on natural focal diseases.

### Data collection

We collected the reported cases of natural focal diseases (brucellosis, malaria, hemorrhagic fever with renal syndrome [HFRS], dengue, severe fever with thrombocytopenia syndrome [SFTS], rabies, tsutsugamushi and Japanese encephalitis [JE]) in 2015–2020 and data on the confirmed COVID-19 cases from January 22, 2020, to June 10, 2021, in Jiangsu Province from the health records of the Jiangsu Provincial Center for Disease Control and Prevention (Jiangsu Provincial CDC). The confirmed cases of COVID-19 were diagnosed based on the World Health Organization (WHO) interim guidance criteria [22]. This study was approved by the Ethical Committee of the Jiangsu Provincial CDC. All data analyzed were anonymized.

### ARIMA model fitting and prediction

Due to the seasonal characteristics of natural focal diseases in Jiangsu Province (except for dengue), an ARIMA (*p*, *d*, *q*) × (*P*, *D*, *Q*)_*s*_ model was adopted for natural focal diseases, and an ARIMA (*p*, *d*, *q*) model for dengue, where AR indicates autoregressive, MA is moving average, *p* and *q* are autoregressive and moving average orders, *P* and *Q* are seasonal autoregressive and moving average orders, *d* is ordinary difference order, *D* is seasonal difference order and *s* is the seasonal cycle.

We drew a time series sequence of the disease, performed seasonal decomposition, autocorrelation and partial autocorrelation analysis on the time series sequence of data, and analyzed the randomness and stationarity of the time series sequence. We used the autocorrelation and partial autocorrelation features to identify the model, test the significance of the established model parameters, and analyze the residuals between the value of the model fitted and reported. We plotted autocorrelation function (ACF) diagrams and partial autocorrelation function (PACF) diagrams for the residual sequence, and used the Ljung–Box residual white noise test to determine whether the residual sequence was a white noise sequence: if *P* > 0.05, then the residual was a white noise sequence, indicating that the established model was suitable and could be used for prediction.

### Statistical analysis

The data were entered into Microsoft Excel 2019 (Microsoft Corp., USA). Nonparametric tests were used to analyze the difference between the reported incidence and the predicted incidence in 2020, difference between reported incidence in 2020 and the previous 4 years, and the difference between the duration from illness onset date to diagnosed date (DID) in 2020 and in the previous 4 years. The determination coefficient (*R*^2^) was used to evaluate the goodness of fit of the model simulation. Statistical analyses and establishment of ARIMA model were conducted with SPSS 13.0 (IBM Corp., Armonk, NY, USA) and GraphPad Prism 7.0 (GraphPad Software, La Jolla, CA, USA) software. A value of *P* < 0.05 indicated that the difference was statistically significant.

## Results

### Incidence trend and curve fitting result for natural focal diseases

**Fig. 1:**
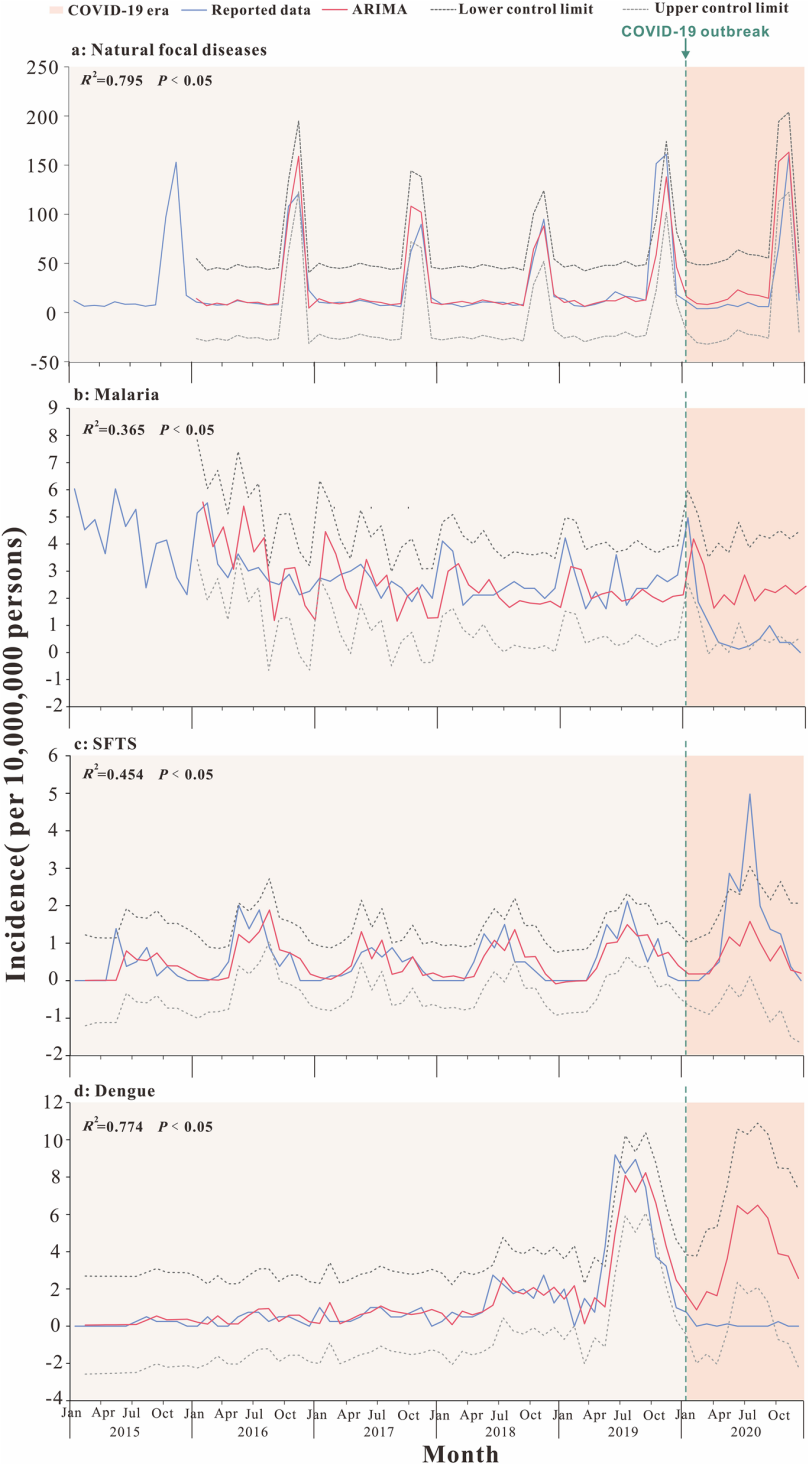
Incidence trend and curve fitting results for natural focal disease, malaria, SFTS and dengue. Jiangsu Province, China, 2015–2020. **a** Natural focal diseases; **b** malaria; **c** SFTS; **d** dengue

From 2015 to 2020, natural focal diseases in Jiangsu Province followed a seasonal trend. The incidence of natural focal diseases was high between September and December each year, with an increasing trend from September, a peak in November, and a decreasing trend afterward (Fig. 1a).

The ARIMA (1,0,0) (0,1,0)_12_ model was constructed after the model parameter test, white noise test (Table 1) and 95% confidence interval (CI) calculation. *R*^2^ = 0.795 and *P* < 0.05, indicated that the model fitted the incidence trends in 2015 to 2019 well, and could be used to predict the incidence of natural focal diseases in Jiangsu Province in 2020. As shown in Fig. 1a, the reported incidence of natural focal diseases in 2020 was lower than the predicted incidence, and the difference was statistically significant (*U* = 31, *P* < 0.05).

**Table 1:** ARIMA model parameters and white noise test of natural focal diseases, malaria, SFTS and dengue

Disease	Model and parameter test						White noise test	
	Model	Parameter	Estimates	Std Error	*t*	*P*	Ljung–Box	*P*
Natural focal diseases	ARIMA (1,0,0) (0,1,0)_12_	AR Non-seasonal lag 1	0.438	0.132	3.326	< 0.05	0.185	0.667
		Seasonal difference	1					
Malaria	ARIMA (1,0,1) (1,1,0)_12_	AR Non-seasonal lag 1	0.97	0.05	19.01	< 0.05	0.089	0.765
		MA Non-seasonal lag 1	0.79	0.12	6.37	< 0.05		
		AR Seasonal lag 1	−0.44	0.14	−3.18	< 0.05		
		Seasonal difference	1					
SFTS	ARIMA (0,1,1) (1,0,0)_12_	AR Non-seasonal lag 1	0.78	0.21	3.67	< 0.05	0.304	0.581
		AR Non-seasonal lag 1	0.98	0.22	4.47	< 0.05		
		Non-seasonal difference	1					
Dengue	ARIMA (1,1,1)	MA Non-seasonal lag 1	0.44	0.12	3.70	< 0.05	1.171	0.279
		AR Seasonal lag 1	0.64	0.11	6.02	< 0.05		
		Non-seasonal difference	1					

ARIMA: autoregressive integrated moving average; AR: autoregressive; MA: moving average; SFTS: severe fever with thrombocytopenia syndrome

### Monthly distribution of natural focal diseases

**Fig. 2:**
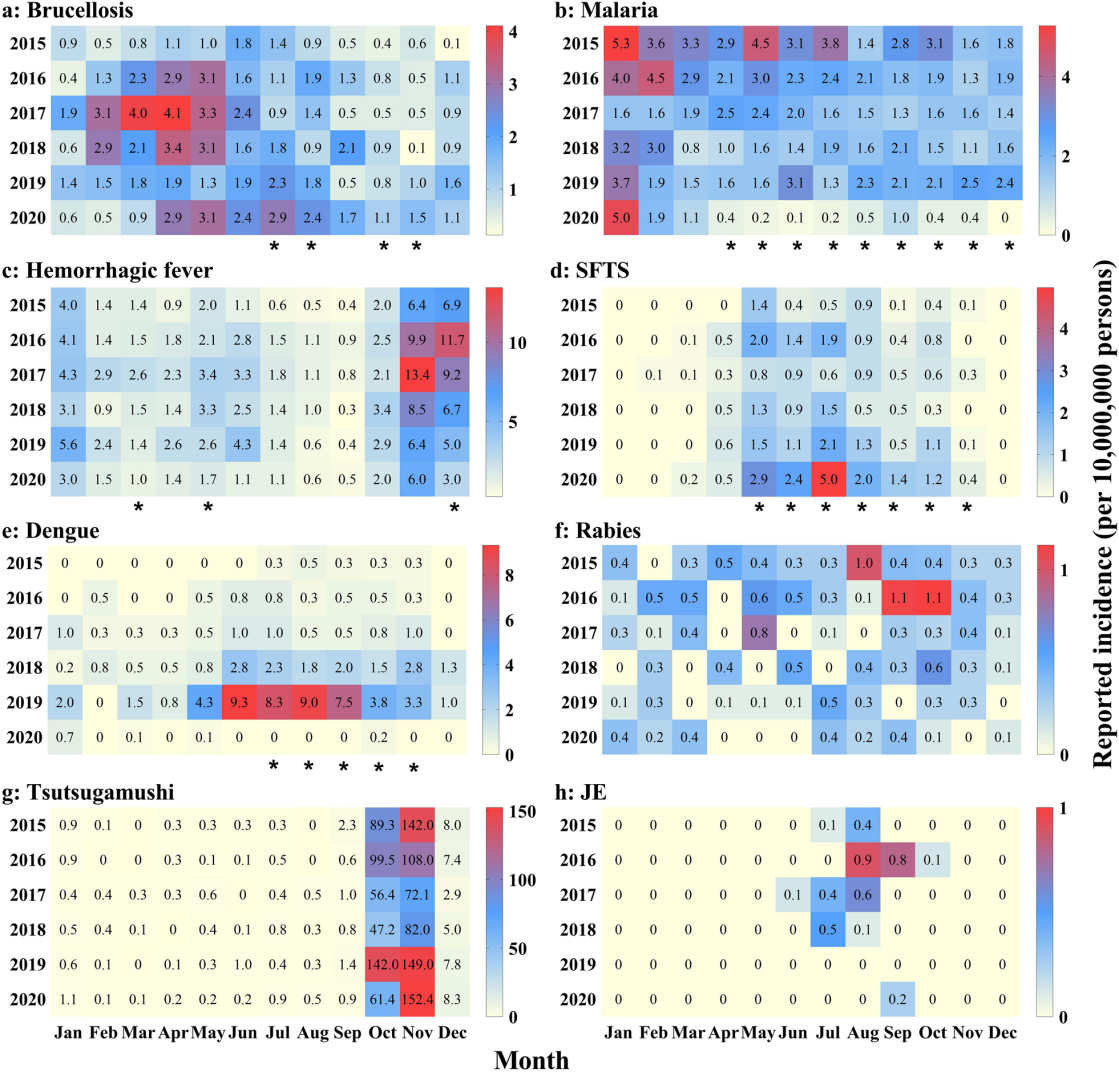
Month distribution of natural focal diseases, Jiangsu Province, China, 2015–2020. **a** Brucellosis; **b** malaria; **c** HFRS; **d** SFTS; **e** dengue; **f** rabies; **g** tsutsugamushi; **h** JE

The monthly incidence in 2020 was compared with that for the period from 2015 to 2019 (Fig. 2). The results showed that the incidence of brucellosis in July, August, October and November 2020 (Fig. 2a) and SFTS in May to November 2020 was higher than that in the same months in the previous 4 years (*P* < 0.05) (Fig. 2d). The incidence of malaria in April to December 2020 (Fig. 2b), HFRS in March, May and December 2020 (Fig. 2d) and dengue in July to November 2020 was lower than that in the same months in the previous 4 years (*P* < 0.05) (Fig. 2e). No statistically significant differences were found in the monthly incidence of other natural focal diseases.

### Incidence trends and curve fitting results for malaria, SFTS and dengue

The incidence of malaria and SFTS in Jiangsu Province followed a long-term seasonal trend. The incidence of dengue was relatively low in 2015–2017, and has increased since 2018. The peak incidence of dengue in 2018 and 2019 was concentrated in June to October.

For malaria, the ARIMA (1,0,1) (1,1,0)_12_ model was constructed after the model parameter test, white noise test (Table 1) and 95% CI calculation. *R*^2^ = 0.365 and *P* < 0.05 indicated that the model fitted the incidence trends in 2015–2019 well, and could be used to predict the incidence of malaria in Jiangsu Province in 2020. As shown in Fig. 1b, the reported incidence in 2020 was lower than the predicted incidence (*U* = 14, *P* < 0.05).

As for SFTS, the ARIMA (0,1,1) (1,0,0)_12_ model was constructed after the model parameter test, white noise test (Table 1) and 95% CI calculation. *R*^2^ = 0.454 and *P* < 0.05 indicated that the model fitted the incidence trends in 2015–2019 well, and could be used to predict the incidence of SFTS in Jiangsu Province in 2020. As shown in Fig. 1c, the reported incidence in 2020 was higher than the predicted incidence (*U* = 68, *P* > 0.05).

For dengue, the ARIMA (1,1,1) model was constructed after model parameter test, white noise test (Table 1) and 95% CI. *R*^2^ = 0.774 and *P* < 0.05 indicated that the model fitted the incidence trends in 2015–2019 well, and could be used to predict the incidence of dengue in Jiangsu Province in 2020. As shown in Fig. 1d, the reported incidence in 2020 was lower than the predicted incidence (*U* = 1, *P* < 0.05).

### Gender distribution of natural focal diseases

**Fig. 3:**
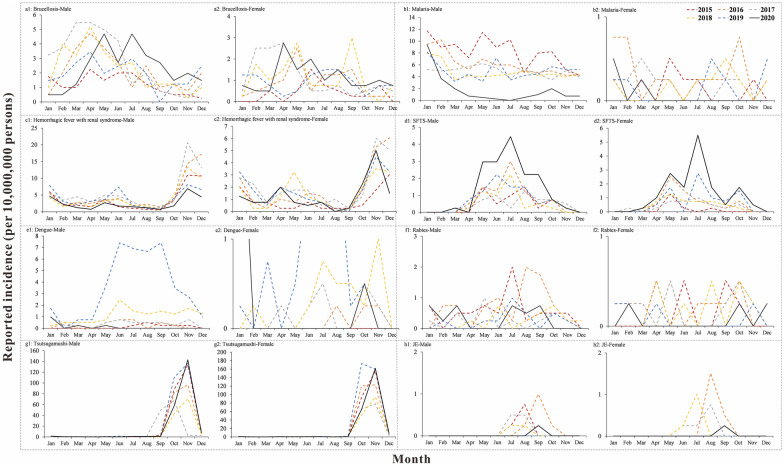
Gender distribution of natural focal diseases, Jiangsu Province, China, 2015–2020. **a** Brucellosis; **b** malaria; **c** HFRS; **d** SFTS; **e** dengue; **f** rabies; **g** tsutsugamushi; **h** JE

For malaria, in males, the incidence in 2020 was lower than in the previous 4 years (*P* < 0.05) (Fig. 3b1), while in females, the incidence in 2020 was lower than in 2016 (*P* < 0.05) (Fig. 3b2). As for SFTS, in females, the incidence in 2020 was higher than in 2015 (*P* < 0.05) (Fig. 3d2). For dengue, in males, the incidence in 2020 was lower than in 2017, 2018 and 2019 (*P* < 0.05) (Fig. 3e1). The incidence of other natural focal diseases in males and females in 2020 was not statistically different from that in the previous 4 years (*P* > 0.05).

### Age distribution of natural focal diseases

**Fig. 4:**
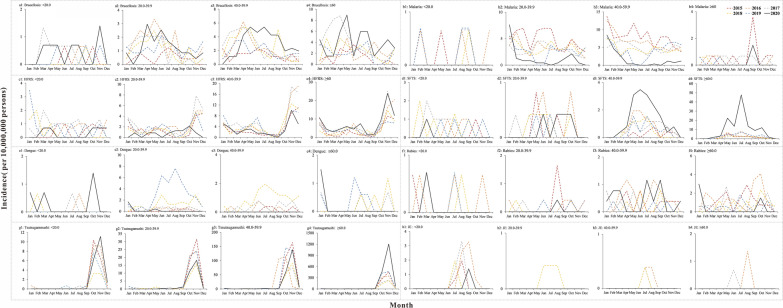
Age distribution of natural focal diseases, Jiangsu Province, China, 2015–2020. **a** Brucellosis; **b** malaria; **c** HFRS; **d** SFTS; **e** dengue; **f** rabies; **g** tsutsugamushi; **h** JE

For malaria, in the 20–60-year age groups, the incidence in 2020 was lower than that in the previous 4 years (*P* < 0.05) (Fig. 4b2, b3).

For SFTS, in the 40–60-year age group, the incidence in 2020 was higher than that in 2015 and 2018 (*P* < 0.05) (Fig. 4d3). In the ≥ 60-year age group, the incidence in 2020 was higher than that in 2015 (*P* < 0.05) (Fig. 4d4).

For dengue, in the 20–40-year age group, the incidence in 2020 was lower than that in 2018 and 2019 (*P* < 0.05) (Fig. 4e2). In the 40–60-year age group, the incidence in 2020 was lower than that in 2016, 2017 and 2018 (*P* < 0.05) (Fig. 4e3).

The incidence of other natural focal diseases in each age group in 2020 was not statistically different from that in the previous 4 years (*P* > 0.05).

### Distribution of natural focal diseases in urban and rural areas

**Fig. 5:**
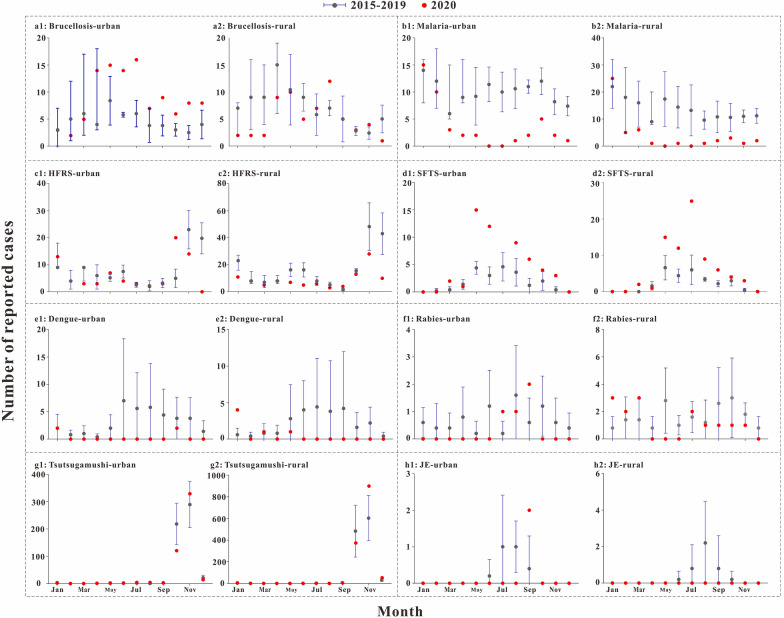
Urban and rural distribution of natural focal diseases, Jiangsu province, China, 2015–2020. **a** Brucellosis; **b** malaria; **c** HFRS; **d** SFTS; **e** dengue; **f** rabies; **g** tsutsugamushi; **h** JE

For brucellosis (Fig. 5a1), the number of cases in 2020 was higher than in 2015, 2016 and 2019 in urban areas (*P* < 0.05).

For malaria (Fig. 5b1, b2), in both urban and rural areas, the number of cases in 2020 was lower than in the previous 4 years (*P* < 0.05).

The number of cases of other natural focal diseases in urban and rural areas in 2020 was not statistically different from that in the previous 4 years (*P* > 0.05).

### Distribution of the DID for natural focal diseases

The DID of brucellosis and SFTS in 2020 was shorter than that in 2015–2018; the DID of tsutsugamushi in 2020 was shorter than that in the previous 4 years (*P* < 0.05). The DID of other natural focal diseases in 2020 was not statistically different from that in the previous 4 years (*P* > 0.05) (Table 2).

**Table 2:** Distribution of duration from illness onset date to diagnosed date for natural focal diseases, Jiangsu Province, China, 2015–2020

Year	Median (IQR) (days)							
	Brucellosis	Malaria	HFRS	SFTS	Dengue	Rabies	Tsutsugamushi	JE
2015	16 (7–35)*	2 (1–4)	6 (4–9)	12 (8–16)*	5 (3–7)	3 (2–4)	5 (2–9)*	24 (7–42)
2016	17 (4–31)*	3 (2–5)	6 (4–10)	14(9–19)*	6 (4–14)	3 (2–5)	5 (2–9)*	38 (11–56)
2017	15 (7–30)*	3 (2–6)	6 (4–10)	11 (7–20)*	7 (5–9)	3 (2–5)	6 (3–9)*	29 (18–79)
2018	10 (4–27)*	3 (2–5)	7 (3–11)	9 (7–14)*	7 (4–10)	3 (2–4)	6 (2–9)*	76 (7–85)
2019	8 (1–16)	3 (2–5)	7 (3–10)	8 (6–12)	6 (4–8)	3 (1–6)	5 (2–8)*	–
2020	7 (1–20)	3 (2–5)	6 (2–11)	8 (4–12)	6 (4–10)	5 (3–6)	5 (2–8)	9 (8–10)

IQR: Interquartile range; HFRS: Hemorrhagic fever with renal syndrome; SFTS: Severe fever with thrombocytopenia syndrome; JE: Japanese encephalitis

*P values have statistical significance

## Discussion

A kind of pneumonia of unknown cause occurred in Wuhan, Hubei, China in December 2019 [23], and was named COVID-19 on February 11, 2020 [24] by WHO. Following the confirmation of the first case of COVID-19 in Jiangsu Province, the provincial government has been working and focusing on epidemic prevention and control. Appropriate countermeasures including restriction of population mobility and lockdown have been taken; thus the number of daily confirmed new cases peaked and then declined rapidly over time in Jiangsu Province [25]. This indicates that the public preventive measures in limiting transmission of COVID-19 were highly effective. Moreover, these measures also had an impact on other diseases [26–28].

In this study, we established ARIMA models to fit and predict the incidence of natural focal diseases, including comprehensive consideration of various factors to select the optimal model, and then used the optimal model to predict incidence in 2020. We found that the reported incidence of natural focal diseases (including brucellosis, malaria, HFRS, dengue, SFTS, rabies, tsutsugamushi and JE) in 2020 was lower than the predicted incidence; the decreased incidence of natural focal diseases may involve other factors: (1) After the outbreak of COVID-19, the number of people who took the initiative to see a doctor may have decreased, resulting in a reduction in reported incidence. (2) Surveillance, reporting and testing of some diseases during COVID-19 were severely affected due to the overburdened health care system. (3) The total incidence of natural focal diseases itself had declined. In future studies, we should consider how to use more direct evidence to evaluate the effect of prevention and control measures taken for COVID-19. We also found that the DID of brucellosis, SFTS and tsutsugamushi in 2020 was shorter than that in the previous years, indicating that during the COVID-19 epidemic, the surveillance efficiency of some natural focal diseases in Jiangsu Province was enhanced. It has been reported that COVID-19 imposed a serious burden on the public health system of many countries, but the positive effects of prevention and control measures on other diseases should not be neglected [29].

The results showed that the reported incidence of brucellosis in July, August, October and November 2020 was higher than that in the same months in the previous 4 years. A study showed that brucellosis is mainly transmitted through contact with sheep or goats [30]. Therefore, during the second half of 2020, with the resumption of routine work, pastoralists had more opportunity to have contact with livestock; thus the probability of infection increased. The reported incidence of malaria in April to December 2020 and dengue in July to November 2020 was lower than that in the same period in 2015–2019. In addition, the incidence of malaria in 2020 changed more sharply compared with that in 2015–2019. The incidence of dengue followed an upward trend in 2015–2019 but declined in 2020. The ARIMA model showed that the reported incidence of malaria and dengue in 2020 was significantly lower than the predicted incidence. The trend in malaria incidence was inconsistent with what was found in other areas or countries [31]. The reason might be that malaria was predominantly endemic in these countries and was mainly caused by the bite of mosquitoes, but in Jiangsu Province, all the malaria cases were imported cases from abroad [32]. The Infectious Disease Surveillance System of the Chinese CDC shows that the cases of dengue in Jiangsu Province were all imported cases from abroad; therefore, travel restrictions could directly reduce the reported cases of dengue and malaria. Furthermore, suspension of work and production might have led to the reduction in reported cases of brucellosis. We also found that the reported incidence of SFTS in 2020 was higher than the predicted incidence, but the difference was not statistically significant; the reason and mechanism need to be further studied.

We next found that the reported incidence of malaria in the 20–60-year age group was lower than that in the previous 4 years. This might be because most of the immigrants into Jiangsu are workers, so the incidence of malaria in this age group was significantly affected by COVID-19 prevention and control measures. The results also showed that the reported incidence of dengue in the 40–60-year age group was lower than that in 2016–2018. This might be because the cases of dengue are mainly in the 20–60-year age group, which is more likely to be affected by the population migration.

Another interesting finding was a lower incidence of dengue in males in 2020 than in 2017–2019. According to the surveillance data, the incidence of dengue fever in males is much higher than in females; therefore, males are more likely to be affected by populational migration. The incidence of malaria in males in 2020 was lower than that in 2015–2019. The majority of imported malaria cases in Jiangsu are males [32], so the incidence of malaria in males in 2020 might be greatly affected by the decrease in imported cases. In addition, we found that the reported incidence of malaria in both urban and rural areas was lower than that in 2015–2019, indicating that the incidence of malaria was affected regardless of the overall incidence, gender, age, or urban or rural area.

## Limitations

Firstly, we are limited in our ability to pinpoint the real reasons for the decrease in natural focal diseases. In future studies, we should consider how to use more direct evidence to show that the change in incidence is caused by the prevention and control measures taken during the COVID-19 pandemic. Secondly, other factors, such as climatic and economic factors, which might affect the spread of the disease [33, 34] were not considered in this study. However, in our research, we established a model to fit the incidence of disease from 2015 to 2019, used the optimal model to predict the incidence of 2020, and then compared the actual incidence before reaching a conclusion. Therefore, the factors mentioned above likely had little influence on our conclusions.

## Conclusions

The interventions for COVID-19 may have had preventative effects on some natural focal diseases in Jiangsu Province. The reported incidence of natural focal diseases, especially malaria and dengue, decreased during the spread of COVID-19 in 2020. COVID-19 prevention and control measures including lockdown, travel restrictions and suspension of work had the greatest impact on males and people aged 20–60 years. The incidence of malaria was affected regardless of the overall incidence, gender, age, or urban or rural area.

## Data Availability

Data supporting the conclusions of this article are included within the article.

## Abbreviations

CDC: Center for Disease Control and Prevention
COVID-19: Coronavirus Disease 2019
HFRS: Hemorrhagic fever with renal syndrome
SFTS: Severe fever with thrombocytopenia syndrome
JE: Japanese encephalitis
ARIMA: Autoregressive integrated moving average
